# Efficient Synthesis of β-Enaminones and β-Enaminoesters Catalyzed by Gold (I)/Silver (I) under Solvent-Free Conditions

**DOI:** 10.3390/molecules17032812

**Published:** 2012-03-06

**Authors:** Ming Zhang, Ablimit Abdukader, Yong Fu, Chengjian Zhu

**Affiliations:** 1 State Key Laboratory of Coordination Chemistry, School of Chemistry and Chemical Engineering, Nanjing University, Nanjing 210093, China; Email: chemliu13@gmail.com (M.Z.); dg1024002@smail.nju.edu.cn (A.A.); fuyong2008@163.com (Y.F.); 2 School of Chemistry and Chemical Engineering, Xinjiang University, Urumqi 830046, China; 3 State Key Laboratory of Organometallic Chemistry, Shanghai Institute of Organic Chemistry, Chinese Academy of Sciences, Shanghai 200032, China

**Keywords:** gold, solvent-free, amines, β-enaminones, β-enaminoesters

## Abstract

An efficient method for the synthesis of β-enaminones and β-enaminoesters using a combination of [(PPh_3_)AuCl]/AgOTf as catalyst has been developed. The reaction between 1,3-dicarbonyl compounds and primary amines was carried out under solvent-free conditions with low catalyst loading in good to excellent yields at room temperature.

## 1. Introduction

β-Enaminones and β-enaminoesters are highly useful building blocks [1,2,3], which can be further transformed into valuable natural therapeutic and biologically active compounds such as anticonvulsivant [4,5], anti-inflammatory [6], and antitumor agents [7,8]. Moreover, they are useful intermediates for the preparation of aminoesters [9], α,β-aminoacids [10,11], peptides [12], quinolines [13,14], azocompounds [15,16] and alkaloids [17,18,19]. Owning to their significances in organic synthesis, considerable efforts have been dedicated to prepare β-enaminones and β-enamino-esters. One of the most straightforward methods is condensation between 1,3-dicarbonyls and amines under reflux conditions [20]. Other improved methods for this amination reaction were successively developed [21,22,23,24,25,26,27,28,29,30,31,32,33,34]. However, in these procedures, the long reaction time, high reaction temperatures, and high catalyst loadings required could limit their further applications in organic synthesis.

**Scheme 1:**
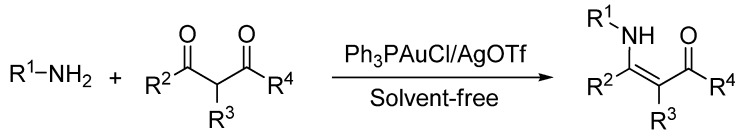
Gold (I)/silver (I) catalyzed enamination of β-dicarbonyl compounds.

Gold (I) and gold (III) salts have emerged as versatile catalysts to facilitate new carbon-carbon or carbon-heteroatom bond formation in a variety of reactions [35,36,37,38]. In 2003, Arcadi reported that gold (III) derivatives could catalyze the condensation reaction of 1,3-dicarbonyls and ammonia/amines [27], however, when the aromatic amine had been involved in the reaction only 60% yield was obtained. With increasing attention being paid to economically simple and environmentally safe methods, the recent trends in organic reaction are oriented to solvent-free conditions [39,40,41]. Herein, we report a practical method for the synthesis of β-enaminones and β-enaminoesters under solvent-free conditions by using [(PPh_3_)AuCl]/AgOTf as catalyst with lower catalyst loading at room temperature (Scheme 1).

## 2. Results and Discussion

Initially, the reaction between acetylacetone and 4-methoxyaniline was carried out without catalyst under solvent-free conditions at room temperature for 2 h; only 25% yield of the desired product could be obtained (Table 1, entry 1). The salt (PPh_3_)AuCl (1 mol%) indicated moderate catalytic activity (33% yield) (Table 1, entry 2), while the silver salt AgOTf (1 mol%) afforded a lower yield (28% yield) (Table 1, entry 3). Surprisingly, by combining (PPh_3_)AuCl (1 mol%) with AgOTf (1 mol%) as cocatalyst, the product could be obtained in higher yield (98%) than when a single salt was used (Table 1, entry 4), so (PPh_3_)AuCl/AgOTf was chosen as a promising catalyst for the reaction. The reaction was found to be sluggish when dichloromethane (DCM, 1 mL) was chosen as solvent (Table 1, entry 5). Various amines were examined in this enamination reaction with acetylacetone, and the corresponding β-enaminones were obtained in excellent yields (85%–98%). The results are listed in Table 2.

**Table 1:** Screening of the reaction conditions for the enamination ^a^. 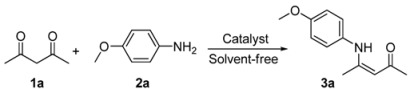

Entry	Catalyst	Time (h)	Yield (%) ^b^
1	-	2	25
2	(PPh_3_)AuCl	2	33
3	AgOTf	2	28
**4**	**(PPh_3_)AuCl + AgOTf**	**2**	**98**
5^c^	(PPh_3_)AuCl + AgOTf	6	85

^a^ Reaction conditions: See typical procedure; ^b^ Isolated yield; ^c^ The reaction was carried out in DCM.

**Table 2:** Synthesis of β-enaminones with [(PPh_3_)AuCl]/AgOTf under solvent-free conditions ^a^. 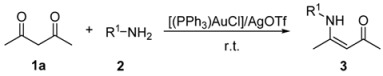

Entry	2 (R^1^)	Time	3	Yield (%) ^b^
1	4-CH_3_OC_6_H_4_	2 h	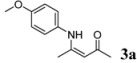	98
2	C_6_H_5_	4 h	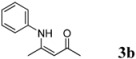	85
3	4-CH_3_C_6_H_4_	3 h	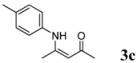	87
4	4-BrC_6_H_4_	4 h	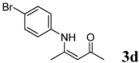	90
5	4-ClC_6_H_4_	5 h	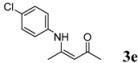	88
6	C_10_H_7_	5 h	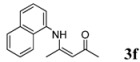	96
7	Bn	5 min	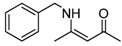 **3g**	95
8	n-C_4_H_9_	5 min	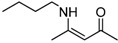 **3h**	96
9	Allyl	1.5 h	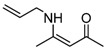 **3i**	98
10	2-Hydroxyethyl	5 min	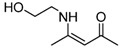 **3j**	96

^a^ Reaction conditions: See typical procedure; ^b^ Isolated yield.

We then extended the scope of various amines with β-ketoesters using 1 mol% loading of [(PPh_3_)AuCl]/AgOTf catalyst, and the results are summarized in Table 3. All the desired products could be obtained in high yields (76–98%). In addition, high chemoselectivity and regioselectivity can be obtained in this reaction since the ester group is less electrophilic than the keto carbonyl group; and only a single product was observed when the reaction was carried out between amine and β-ketoesters. Generally, the electronic properties of the aryl group appeared to slightly influence the reactivity. It is clear from our results that aromatic amines containing electron-donating groups like methoxyl and methyl (Table 3, entries 2 and 3) give the corresponding products in higher yields compared to the electron-withdrawing ones (Table 3, entries 4 and 5). Aliphatic amines (Table 3, entries 7–10) were more reactive than aromatic amines (Table 3, entries 1–6), and the reactions were completed within a shorter time. Furthermore, cyclic β-ketoesters could also afford the desired product in high yields (Table 3, entries 11–16). 

**Table 3:** Synthesis of β-enaminoesters with [(PPh_3_)AuCl]/AgOTf under solvent-free conditions ^a^. 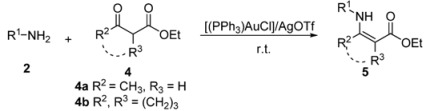

Entry	2 (R^1^)	4	Time	5	Yield (%) ^b^
1	4-CH_3_OC_6_H_4_	**4a**	3 h	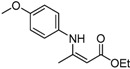 **5a**	98
2	C_6_H_5_	**4a**	5 h	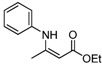 **5b**	82
3	4-CH_3_C_6_H_4_	**4a**	4 h	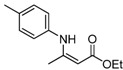 **5c**	92
4	4-BrC_6_H_4_	**4a**	5 h	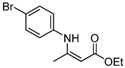 **5d**	86
5	4-ClC_6_H_4_	**4a**	5 h	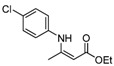 **5e**	76
6	C_10_H_7_	**4a**	8 h	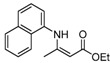 **5f**	85
7	Bn	**4a**	5 min	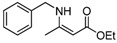 **5g**	97
8	n-C_4_H_9_	**4a**	5 min	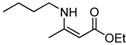 **5h**	95
9	Allyl	**4a**	1 h	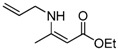 **5i**	97
10	2-Hydroxyethyl	**4a**	5 min	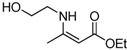 **5j**	96
11	4-CH_3_OC_6_H_4_	**4b**	2 h	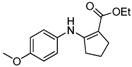 **5k**	93
12	C_6_H_5_	**4b**	2 h	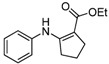 **5l**	87
13	4-CH_3_C_6_H_4_	**4b**	1.5 h	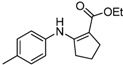 **5m**	94
14	4-BrC_6_H_4_	**4b**	2 h	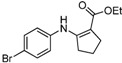 **5n**	92
15	4-ClC_6_H_4_	**4b**	2 h	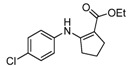 **5o**	90
16	C_10_H_7_	**4b**	2 h	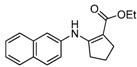 **5p**	90
17	Bn	**4b**	5 min	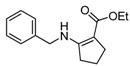 **5q**	85

^a^ Reaction conditions: See typical procedure; ^b^ Isolated yield.

## 3. Experimental

### 3.1. General

All reagents were obtained from commercial suppliers and used without further purification. Solvents were dried and distilled prior to use according to standard methods. The reaction was monitored by TLC on silica-gel plates (GF 254). ^1^H- (300 MHz) and ^13^C-NMR (75 MHz) spectra were recorded on a Bruker APX-300 spectrometer at room temperature in CDCl_3_ using tetramethylsilane (TMS) as the internal standard. The coupling constants *J *are given in Hz. All yields mentioned referred to isolated yields. 

### 3.2. Preparation of (PPh_3_)AuCl

The reaction was carried out in the absence of light. SMe_2_ (10 mmol) was added to a solution of HAuCl_4_·2H_2_O (5 mmol) in MeOH (5 mL), and the mixture was stirred for 10 minutes. The white precipitate [AuCl(SMe_2_)] from solution was then filtered, subsequently washed with MeOH, Et_2_O and hexane, then dried under vacuum and used in the next step without further purification. Triphenylphosphine (2 mmol) was added to a stirred solution of [AuCl(SMe_2_)] (2 mmol) in CH_2_Cl_2_ (15 mL) under a nitrogen atmosphere. After stirring at room temperature for 30 minutes, the volume of the solution was reduced to 5 mL under reduced pressure, and then hexane (20 mL) was added, resulting in the precipitation of the complex. The solid was then filtered, washed with hexane and dried, resulting in the quantitative isolation of complex [(PPh_3_)AuCl] as a pale yellow solid (97%). The complex was characterized only by ^31^P-NMR spectroscopy [42,43].

### 3.3. Typical Procedure for the Synthesis of β-Enaminones and β-Enaminoesters

The reaction was carried out without inert atmosphere and light protection. A mixture of (PPh_3_)AuCl (0.03 mmol), AgOTf (0.03 mmol) and 1,3-dicarbonyl compound (3 mmol) was stirred at room temperature for 5 minutes, then amine (3 mmol) was added into the stirring solution. The reaction was monitored by TLC on silica-gel plates (GF 254). After the reaction was complete, the residue was diluted with water (10 mL) and extracted with ethyl acetate (3 × 10 mL). The combined organic extracts were dried over anhydrous Na_2_SO_4_ and concentrated under reduced pressure after filtration. Further purification by flash chromatography gave the corresponding product.

*(Z)-4-(4-Methoxyphenylamino)pent-3-en-2-one* (**3a**). ^1^H-NMR δ = 12.31 (s, 1H), 7.03–7.05 (m, 2H), 6.86–6.88 (m, 2H), 5.16 (s, 1H), 3.79 (s, 3H), 2.08 (s, 3H), 1.90 (s, 3H); ^13^C-NMR δ = 195.78, 161.15, 157.60, 131.35, 126.54, 114.13, 96.79, 55.33, 28.99, 19.54.

*(Z)-4-(Phenylamino)pent-3-en-2-one* (**3b**). ^1^H-NMR δ = 12.44 (s, 1H), 6.96–7.23 (m, 5H), 5.09 (s, 1H), 1.99 (s, 3H), 1.87 (2, 3H); ^13^C-NMR δ = 195.45, 159.62, 138.18, 128.59, 124.98, 124.05, 97.18, 28.66, 19.30

*(Z)-4-(p-Tolylamino)pent-3-en-2-one* (**3c**). ^1^H-NMR δ = 12.39 (s, 1H), 7.07 (d, 2H, *J* = 8.4 Hz), 6.92 (d, 2H, *J* = 8.4 Hz), 5.12 (s, 1H), 2.27 (s, 3H), 2.04 (s, 3H), 1.89 (s, 3H); ^13^C-NMR δ = 195.67, 160.51, 135.91, 135.27, 129.54, 124.62, 97.13, 28.98, 20.77, 19.61

*(Z)-4-(4-Bromophenylamino)pent-3-en-2-one* (**3d**). ^1^H-NMR δ = 12.38 (s, 1H), 7.34 (d, 2H, *J* = 12 Hz), 6.88 (d, 2H, *J* = 12 Hz), 5.13 (s, 1H), 2.01 (s, 3H), 1.90 (s, 3H); ^13^C-NMR δ = 196.31, 159.32, 137.74, 132.02, 125.83, 118.43, 98.16, 29.13, 19.70.

*(Z)-4-(4-Chlorophenylamino)pent-3-en-2-one* (**3e**). ^1^H-NMR δ = 12.38 (s, 1H), 7.18–7.22 (m, 2H), 6.92–6.96 (m, 2H), 5.12 (s, 1H), 2.01 (s, 3H), 1.89 (s, 3H); ^13^C-NMR δ = 196.3, 159.5, 137.2, 129.1, 125.6, 98.0, 29.1, 19.7.

*(Z)-4-(Naphthalen-1-ylamino)pent-3-en-2-one* (**3f**). ^1^H-NMR δ = 12.80 (s, 1H), 8.02–8.05 (m, 1H), 7.83–7.86 (m, 1H), 7.72–7.75 (m, 1H), 7.49–7.55 (m, 2H), 7.39–7.44 (m, 1H), 7.23–7.26 (m, 1H), 5.31 (s, 1H), 2.12 (s, 3H), 1.85 (s, 3H); ^13^C-NMR δ = 196.4, 161.8, 134.7, 134.1, 129.8, 128.2, 126.8, 126.7, 126.5, 125.2, 123.3, 122.6, 97.5, 29.2, 19.6.

*(Z)-4-Benzylamino)pent-3-en-2-one* (**3g**). ^1^H-NMR δ = 11.17 (s, 1H), 7.22–7.34 (m, 5H), 5.03 (s, 1H), 4.44 (d, 2H, *J* = 6.3 Hz), 2.02 (s, 3H), 1.88 (2, 3H); ^13^C-NMR δ = 195.1, 162.9, 137.8, 128.6, 127.2, 126.5, 95.7, 77.4, 77.0, 76.6, 46.4, 28.7, 18.7.

*(Z)-4-(Butylamino)pent-3-en-2-one* (**3h**). ^1^H-NMR δ = 10.80 (s, 1H), 4.87 (s, 1H), 3.15 (q, 2H, *J* = 6.6 Hz), 1.91 (s, 3H), 1.84 (s, 3H), 1.44–1.54 (m, 2H), 1.27–1.39 (m, 2H), 0.85 (q, 3H, *J* = 7.2 Hz); ^13^C- NMR δ = 194.3, 162.9, 94.7, 42.4, 31.9, 28.5, 19.8, 19.7, 18.6, 13.5, 13.5.

*(Z)-4-(Allylamino)pent-3-en-2-one* (**3i**). ^1^H-NMR δ = 10.70 (s, 1H), 5.64–5.73 (m, 2H), 4.97–5.07 (m, 2H), 4.83 (s, 3H), 3.67–3.71(m, 2H), 1.82 (s, 1H), 1.74(s, 1H); ^13^C-NMR δ = 194.7, 162.9, 133.9, 115.7, 95.4, 44.8, 28.6, 18.3.

*(Z)-4-(2-Hydroxyethylamino)pent-3-en-2-one* (**3j**). ^1^H-NMR δ = 10. 71 (s, 1H), 5.02 (s, 1H), 4.83 (s, 1H), 3.60 (t, 2H, *J* = 5.4 Hz), 3.27 (t, 2H, *J* = 5.4 Hz), 1.83 (s, 6H); ^13^C-NMR δ = 194.3, 164.1, 95.3, 61.0, 45.3, 28.3, 18.9.

*(Z)-Ethyl 3-(4-Methoxyphenylamino)but-2-enoate* (**5a**). ^1^H-NMR δ = 10.15 (s, 1H), 6.97–7.00 (m, 2H), 6.80–6.83 (m, 2H), 4.62 (s, 1H), 4.07–4.14 (q, 2H, *J* = 7.0 Hz), 3.75 (s, 3H), 1.85 (s, 3H), 1.24 (t, 3H, *J* = 7.0 Hz); ^13^C-NMR δ = 170.3, 159.7, 157.2, 131.9, 126.5, 113.9, 84.5, 58.4, 55.1, 19.9, 14.4.

*(Z)-Ethyl 3-(Phenylamino)but-2-enoate* (**5b**). ^1^H-NMR δ = 10.43 (s, 1H), 7.27–7.32 (m, 2H), 7.05–7.15 (m, 3H), 4.70 (s, 1H), 4.11–4.18 (q, 2H, *J* = 7.2 Hz), 1.97 (s, 3H), 1.27 (t, 3H, *J* = 7.2 Hz); ^13^C-NMR δ = 170.3, 158.8, 139.2, 120.0, 124.8, 124.2, 86.0, 58.6, 20.2, 14.5.

*(Z)-Ethyl 3-(p-Tolylamino)but-2-enoate* (**5c**). ^1^H-NMR δ = 10.32 (s, 1H), 7.10–7.13 (m, 2H), 6.95–6.98 (m, 2H), 4.67 (s, 1H), 4.14 (q, 2H, *J* = 7.2 Hz), 2.32 (s, 3H), 1.94 (s, 3H), 1.28 (t, 3H, *J* = 7.2 Hz). ^13^C-NMR δ = 170.3, 159.3, 136.6, 134.7, 129.5, 124.6, 85.3, 58.6, 20.8, 20.1, 14.5.

*(Z)-Ethyl 3-(4-Bromophenylamino)but-2-enoate* (**5d**). ^1^H-NMR δ = 10.34 (s, 1H), 7.34–7.37 (m, 2H), 6.88–6.89 (m, 2H), 4.67 (s, 1H), 4.06–4.13 (q, 2H, *J* = 7.2 Hz), 1.93 (s, 3H), 1.23 (t, 3H, *J* = 7.2 Hz); ^13^C-NMR δ = 170.2, 158.0, 138.4, 132.0, 125.5, 117.7, 87.0, 58.8, 20.1, 14.5.

*(Z)-Ethyl 3-(4-Chlorophenylamino)but-2-enoate* (**5e**). ^1^H-NMR δ = 10.36 (s, 1H), 7.24–7.27 (m, 2H), 6.97–7.00 (m, 2H), 4.70 (s, 1H), 4.12 (q, 2H, *J* = 7.2 Hz), 1. 95 (s, 3H), 1.26 (t, 3H, *J* = 7.2 Hz). ^13^C-NMR δ = 170.3, 158.2, 137.9, 130.1, 129.1, 125.3, 116.1, 86.8, 58.8, 20.1, 14.5.

*(Z)-Ethyl 3-(Naphthalen-1-ylamino)but-2-enoate* (**5f**). ^1^H-NMR δ = 10.68 (s, 1H), 8.10–8.13 (m, 1H), 7.87–7.90 (m, 1H), 7.74–7.77 (m, 1H), 7.52–7.57 (m, 2H), 7.42–7.47 (m, 1H), 7.27–7.29 (m, 1H), 4.88 (s, 1H), 4.27 (q, 2H, *J* = 7.2 Hz), 1. 89 (s, 3H), 1.37 (t, 3H, *J* = 7.2 Hz). ^13^C-NMR δ = 170.7, 160.4, 135.3, 134.2, 130.4, 128.2, 126.7, 126.5, 126.4, 125.3, 123.5, 122.7, 85.8, 58.8, 20.0, 14.7.

*(Z)Ethyl 3-(Benzylamino)but-2-enoate* (**5g**). ^1^H-NMR δ = 8.96 (s, 1H), 7.21–7.29 (m, 5H), 4.53 (s, 1H), 4.34 (s, 2H), 4.04–4.11 (m, 3H), 1.84 (t, 3H, *J* = 3.6 Hz), 1.18–1.24 (m, 3H); ^13^C-NMR δ = 170.4, 161.7, 138.7, 128.7, 127.2, 126.6, 83.1, 58.2, 46.6, 19.2, 14.5.

*(Z)-Ethyl 3-(Butylamino)but-2-enoate* (**5h**). ^1^H-NMR δ = 8.46 (s, 1H), 4.31 (s, 1H), 3.96 (q, 2H, *J* = 7.2 Hz), 3.06–3.13 (m, 2H), 1.81 (s, 3H), 1.40–1.49 (m, 2H), 1.24–1.36 (m, 2H), 1.13 (t, 3H, *J* = 7.2 Hz), 0.83 (t, 3H, *J* = 7.2 Hz); ^13^C-NMR δ = 170.4, 161.7, 81.5, 57.9, 42.5, 32.3, 19.8, 19.1, 14.4, 13.6.

*(Z)-Ethyl 3-(Allylamino)but-2-enoate* (**5i**). ^1^H NMR (CDCl3, 300 MHz) δ = 8.61 (s, 1H), 5.74–5.85 (m, 1H), 5.06–5.19 (m, 2H), 4.42 (s, 1H), 4.02 (q, 2H, *J* = 7.2 Hz), 3.75–3.80 (m, 2H), 1.85 (s, 3H), 1.18 (t, 3H, *J* = 7.2 Hz); ^13^C NMR (CDCl_3_, 75 MHz) δ = 170.4, 161.7, 134.7, 115.6, 82.6, 58.1, 45.0, 18.9, 14.5.

*(Z)-Ethyl 3-(2-Hydroxyethylamino)but-2-enoate* (**5j**). ^1^H NMR (CDCl_3_, 300 MHz) δ = 8.61 (s, 1H), 4.44 (s, 1H), 4.04 (q, 2H, *J* = 7.2 Hz), 3.70 (t, 2H, *J* = 5.4 Hz), 3.13 (br, 1H), 3.33 (q, 2H, *J* = 5.4 Hz), 1.91 (s, 1H), 1.21 (t, 3H, *J* = 7.2 Hz); ^13^C NMR (CDCl_3_, 75 MHz) δ = 170.7, 162.1, 82.6, 61.8, 58.4, 45.0, 19.5, 14.5.

*Ethyl 2-(4-Methoxyphenylamino)cyclopent-1-enecarboxylate* (**5k**). ^1^H-NMR δ = 9.32 (s, 1H), 6.90–6.93 (m, 2H), 6.74–6.77 (m, 2H) , 4.14 (q, 2H, *J* = 7.2 Hz), 3.69 (s, 1H), 2.49–2.60 (m, 4H), 1.71–1.80 (m, 2H), 1.25 (t, 3H, *J* = 7.2 Hz); ^13^C-NMR δ = 168.3, 161.4, 156.1, 133.6, 123.3, 114.1, 95.9, 58.6, 55.2, 33.1, 28.8, 21.5, 14.6.

*Ethyl 2-(Phenylamino)cyclopent-1-enecarboxylate* (**5l**). ^1^H-NMR δ = 9.65 (s, 1H), 7.24–7.30 (m, 2H), 6.99–7.05 (m, 3H) , 4.21 (q, 2H, *J* = 7.2 Hz), 2.79 (t, 2H, *J* = 7.2 Hz), 2.58 (t, 2H, *J* = 7.2 Hz), 1.86 (m, 2H), 1.32 (t, 3H, *J* = 7.2 Hz); ^13^C-NMR δ = 168.4, 160.3, 140.6, 129.1, 123.0, 120.6, 97.6, 58.9, 33.6, 28.7, 21.7, 14.6.

*Ethyl 2-(p-Tolylamino)cyclopent-1-enecarboxylate* (**5m**). ^1^H-NMR δ = 9.55 (s, 1H), 7.05–7.08 (m, 2H), 6.92–6.94 (m, 2H), 4.21 (q, 2H, *J* = 7.2 Hz), 2.73 (t, 2H, *J* = 7.2 Hz), 2.57(t, 2H, *J* = 7.2 Hz), 2.29 (s, 3H), 1.84 (m, 2H), 1.31(t, 3H, *J* = 7.2 Hz); ^13^C-NMR δ = 168.4, 160.8, 138.1, 132.7, 129.6, 121.0, 96.8, 58.8, 33.4, 28.7, 21.7, 20.6, 14.6.

*Ethyl 2-(4-Bromophenylamino)cyclopent-1-enecarboxylate* (**5n**). ^1^H-NMR δ = 9.61 (s, 1H), 7.30–7.35 (m, 2H), 6.83–6.88 (m, 2H) , 4.11 (q, 2H, *J* = 7.2 Hz), 2.72 (t, 2H, *J* = 7.2 Hz), 2.53 (t, 2H, *J* = 7.2 Hz), 1.83 (m, 2H), 1.28 (t, 3H, *J* = 7.2 Hz); ^13^C-NMR δ = 168.3, 159.4, 139.7, 132.0, 121.7, 115.4, 98.6, 59.0, 33.5, 28.6, 21.7, 14.6.

*Ethyl 2-(4-Chlorophenylamino)cyclopent-1-enecarboxylate* (**5o**). ^1^H-NMR δ = 9.60 (s, 1H), 7.16–7.19 (m, 2H), 6.89–6.92 (m, 2H) , 4.16 (q, 2H, *J* = 7.2 Hz), 2.72 (t, 2H, *J* = 7.2 Hz), 2.53 (t, 2H, *J* = 7.2 Hz), 1.83 (m, 2H), 1.27 (t, 3H, *J* = 7.2 Hz); ^13^C-NMR δ = 168.3, 159.5, 139.2, 129.0, 127.9, 121.4, 98.4, 59.0, 33.5, 28.6, 21.6, 14.5.

*Ethyl 2-(Naphthalen-1-ylamino)cyclopent-1-enecarboxylate* (**5p**). ^1^H-NMR δ = 10.02 (s, 1H), 8.18–8.21 (m, 1H), 7.85–7.88 (m, 1H), 7.52–7.66 (m, 3H), 7.38–7.44 (m, 1H), 7.22–7.25 (m, 1H), 7.27–7.29 (m, 1H), 4.33 (q, 2H, *J* = 7.2 Hz), 2.69 (m, 4H), 1. 86 (m, 2H), 1.40 (t, 3H, *J* = 7.2 Hz); ^13^C-NMR δ = 168.8, 161.6, 136.3, 134.3, 128.3, 126.4, 126.3, 125.4, 124.6, 122.0, 119.0, 97.7, 59.0, 33.4, 29.0, 21.6, 14.8. 

*Ethyl 2-(Benzylamino)cyclopent-1-enecarboxylate* (**5q**). ^1^H-NMR δ = 7.80 (s, 1H), 7.25–7.35 (m, 5H), 4.38 (d, 2H, *J* = 6.6 Hz) , 4.16 (q, 2H, *J* = 7.2 Hz), 2.54 (q, 4H, *J* = 7.2 Hz), 1.81 (m, 2H), 1.28 (t, 3H, *J* = 7.2 Hz); ^13^C-NMR δ = 168.4, 164.5, 139.2, 128.6, 127.2, 126.7, 93.4, 58.4, 48.3, 32.0, 29.1, 20.8, 14.7.

## 4. Conclusions

In summary, we have developed an efficient method for the synthesis of β-enaminones and β-enaminoesters *via* reaction of 1,3-dicarbonyl compounds with various primary amines under solvent-free conditions catalyzed by [(PPh_3_)AuCl]/AgOTf. This methodology affords various β-enaminones and β-enaminoesters derivatives in good to excellent yields. Moreover, the method has the advantage of easy manipulation and mild reaction conditions.
